# Antimicrobial Activity of α-Peptide/β-Peptoid Lysine-Based Peptidomimetics Against Colistin-Resistant *Pseudomonas aeruginosa* Isolated From Cystic Fibrosis Patients

**DOI:** 10.3389/fmicb.2019.00275

**Published:** 2019-02-20

**Authors:** Natalia Molchanova, Hengzhuang Wang, Paul R. Hansen, Niels Høiby, Hanne M. Nielsen, Henrik Franzyk

**Affiliations:** ^1^Department of Drug Design and Pharmacology, Faculty of Health and Medical Sciences, University of Copenhagen, Copenhagen, Denmark; ^2^Department of Clinical Microbiology, Copenhagen University Hospital, Copenhagen, Denmark; ^3^Costerton Biofilm Center, Department of Immunology and Microbiology, Faculty of Health and Medical Sciences, University of Copenhagen, Copenhagen, Denmark; ^4^Department of Pharmacy, Faculty of Health and Medical Sciences, University of Copenhagen, Copenhagen, Denmark

**Keywords:** peptidomimetic, *Pseudomonas aeruginosa*, antimicrobial, cystic fibrosis, resistance

## Abstract

*Pseudomonas aeruginosa* infection is a predominant cause of morbidity and mortality in patients with cystic fibrosis infection and with a compromised immune system. Emergence of bacterial resistance renders existing antibiotics inefficient, and therefore discovery of new antimicrobial agents is highly warranted. In recent years, numerous studies have demonstrated that antimicrobial peptides (AMPs) constitute potent agents against a range of pathogenic bacteria. However, AMPs possess a number of drawbacks such as susceptibility to proteolytic degradation with ensuing low bioavailability. To circumvent these undesired properties of AMPs unnatural amino acids or altered backbones have been incorporated to provide stable peptidomimetics with retained antibacterial activity. Here, we report on antimicrobial α-peptide/β-peptoid lysine-based peptidomimetics that exhibit high potency against clinical drug-resistant *P. aeruginosa* strains obtained from cystic fibrosis patients. These clinical strains possess *phoQ* and/or *pmrB* mutations that confer high resistance to colistin, the last-resort antibiotic for treatment of infections caused by *P. aeruginosa*. The lead peptidomimetic LBP-2 demonstrated a 12-fold improved anti-pseudomonal activity as compared to colistin sulfate as well as favorable killing kinetics, similar antibiofilm activity, and moderate cytotoxicity.

## Introduction

*Pseudomonas aeruginosa* is a nosocomial pathogen that causes severe chronic and acute infections, leading to high morbidity and mortality, especially among immunocompromised patients (Lyczak et al., 2000). For several decades, this bacterium has been the most prevalent respiratory pathogen in patients with cystic fibrosis (CF) (Høiby et al., 2010). Once *P. aeruginosa* has colonized the respiratory tract mucosal epithelium it is almost impossible to eradicate, resulting in a chronic lung infection (Banerjee and Stableforth, 2000). The ability of *P. aeruginosa* to form robust biofilms renders antibiotic treatment inefficient while promoting resistance development (Drenkard, 2003). Continuous intensive antibiotic treatment and high tolerance of biofilms have resulted in the emergence of multidrug-resistant (MDR) *P. aeruginosa* strains (Johansen et al., 2008; Høiby et al., 2010; Mulcahy et al., 2014). Colistin constitutes the last-resort antibiotic against these MDR infections, and hence development of resistance to colistin among CF patients is particularly worrying (Moskowitz et al., 2012). Lipopolysaccharide (LPS) that constitutes the major part of the Gram-negative outer membrane interacts with colistin through electrostatic bonds between its anionic phosphate groups and the protonated amino groups of the highly cationic cyclic lipopeptide (Storm et al., 1977). Colistin resistance and induced colistin tolerance is associated with reduction of the bacterial outer-membrane negative charge conferred by modification of lipid A and the core oligosaccharide with 4-amino-L-arabinose (L-Ara4N) residues, thereby diminishing interactions between colistin and LPS (Moskowitz et al., 2004; Pamp et al., 2008; Lee and Ko, 2014).

In 2016, FDA approved a new combination of two commonly used drugs for treatment of infections caused by *P. aeruginosa*, namely ceftolozane and tazobactam. However, it has not been well studied in cystic fibrosis patients with an MDR *P. aeruginosa* infection (Ang et al., 2016; Haidar et al., 2017; Shortridge et al., 2017). No new classes of antibiotics against Gram-negative bacteria have been introduced for clinical use in recent years (Butler et al., 2017; Fernandes and Martens, 2017), and hence the discovery of alternative potent antimicrobial compounds against Gram-negative pathogens is of crucial importance. This implies that development of new approaches for successful treatment of *P. aeruginosa* infections will alleviate a major health issue.

Antimicrobial peptides (AMPs) play a crucial role in the innate host defense as the frontline endogenous defense molecules that prevent establishment of infections caused by invasive pathogens (Haney et al., 2017; Molchanova et al., 2017a). Within the last decade, AMPs have been demonstrated to possess potency against various susceptible and resistant *P. aeruginosa* strains e.g., several synthetic AMPs were reported to exert antibacterial activity with minimum inhibitory concentrations (MICs) of 1 μg/mL (Lim et al., 2006; Joshi et al., 2010; Shin, 2014; Monteiro et al., 2015). Notably, cecropin displayed MIC and minimum bactericidal concentration (MBC) values of 0.75 and 1.5 μg/mL, respectively (Abou-Zied et al., 2015). In addition, it has been reported that certain AMPs successfully inhibit bacterial biofilm development, and therefore may be used in combination with commonly used antibiotics to prevent biofilm formation as well to eradicate established biofilms (de la Fuente-Nunez and Hancock, 2015; Andrea et al., 2018).

Though AMPs are considered as potential leads for novel antibacterial agents, they generally suffer from several shortcomings, e.g., poor bioavailability due to susceptibility to proteolytic degradation and their often unfavorable hemolytic and cytotoxic properties conferring a narrow therapeutic window (Rotem and Mor, 2009). Incorporation of peptoid residues (i.e., N-alkylated glycine residues) leads to enhanced proteolytic stability while allowing for the introduction of side chains similar to those present in natural α-amino acids as well as a vast number of variations only limited by the availability of suitably protected primary amines (Zuckermann and Kodadek, 2009). In a previous study, an α-peptide/α-peptoid hybrid was found to exhibit potent activity against *P. aeruginosa* demonstrating prevention of biofilm formation (Jahnsen et al., 2013). Additionally, recent structure-activity relationship studies revealed that a subclass of α-peptide/β-peptoid hybrids (e.g., lysine-based peptidomimetic **1** = LBP-1) display broad-spectrum activity against Gram-positive and Gram-negative bacteria (Molchanova et al., 2017b), and that LBP-2 was demonstrated to possess high potency against *P. aeruginosa* (Klodzinska et al., 2018). In the present study, we have designed a few next-generation analogs based on LBP-2 and investigated the activity of this series of compounds against *P. aeruginosa* strains including several resistant strains obtained from CF patients.

## Materials and Methods

### Chemicals and General Procedures for Compound Characterization

Starting materials and solvents were purchased from commercial suppliers (Iris Biotech, Sigma-Aldrich, and Merck) and were used without further purification. H-Rink-Amide ChemMatrix resin (loading 0.52 mmol/g, 0.05 mmol) was purchased from PCAS BioMatrix Inc., QC, Canada. Preparative HPLC was performed by using a Phenomenex Luna C18 (2) column (250 mm × 21.2 mm; 5 μm particle size) on a Shimadzu Prominence system using an aqueous MeCN gradient with 0.1% TFA added (eluent A: 5:95 MeCN - H_2_O with 0.1% TFA added; eluent B: 95:5 MeCN - H_2_O with 0.1% TFA added). Analytical UHPLC was performed by using a Phenomenex Luna C18 (2) HST column (100 mm × 3.0 mm; 2.5 μm particle size) on a Shimadzu Prominence and Shimadzu Nexera system by using the above eluents A and B. All tested compounds had a purity of at least 95%. HRMS spectra were obtained by using a Bruker MicrOTOF-Q II Quadrupole MS detector.

### Manual Synthesis of Peptidomimetics

Peptidomimetics were prepared manually in teflon vessels (10 mL; fitted with a polypropylene filter) using dimeric α-peptide/β-peptoid building blocks as previously described (Bonke et al., 2008). Dimeric building blocks were synthesized as previously reported (Bonke et al., 2008). In brief, 5.0 equiv Fmoc-protected dimeric building block, 5.0 equiv PyBOP (benzotriazol-1-yl-oxytripyrrolidinophosphonium hexafluorophosphate) and 10.0 equiv DIPEA (N,N-diisopropylethylamine) were mixed and added to the resin, which then was shaken at room temperature for at least 2 h. Fmoc deprotection was performed with 20% piperidine in N,N-dimethylformamide (DMF, 2 × 10 min, each time with 5 mL under shaking at room temperature). Each step was followed by washing with DMF, MeOH, and CH_2_Cl_2_ (each for 3 × 3 min, each time with 5 mL under shaking at room temperature). Capping was performed with the mixture Ac_2_O–DIPEA–(N-methyl-2-pyrrolidone) 1:2:3 (5 mL for 10 min under shaking at room temperature) after the loading of the first dimeric building block. Cleavage of the peptidomimetics from the resin and simultaneous side-chain deprotection were carried out by 2 × 1 h treatments with the mixture TFA–TIS–H_2_O (95:2.5:2.5). The crude product was purified by preparative HPLC. For elution of peptidomimetics, a linear gradient of 10% → 60% B during 10 min was used with UV detection at λ = 220 nm. The appropriate fractions were concentrated *in vacuo* and lyophilized; compound identity was verified by HRMS, and purity (>95%) was determined by analytical UHPLC.

### Strain Description

The non-mucoid wild-type *P. aeruginosa* strain PAO1, and its isogenic mucoid variant Alg^+^PAOmucA22 (PDO300) were used as control strains in this study (Hengzhuang et al., 2011). Clinical isolates of *P. aeruginosa* PA1016, PA1603 PA77685, PA44638, and PA41782 were collected from the sputum of CF patients. PA1016 and PA1603 have both *phoQ* and *pmrB* mutations (Miller et al., 2011; Moskowitz et al., 2012). PA77685 and PA44638 have *pmrB* mutation. PA41782 has *phoQ* mutation (Miller et al., 2011; Moskowitz et al., 2012). All selected clinical strains with *phoQ* and/or *pmrB* mutations showed high resistance to colistin.

### Determination of Minimal Inhibitory Concentrations

*Pseudomonas aeruginosa* strain PAO1 and clinical isolates were obtained from Department of Clinical Microbiology, University Hospital of Copenhagen, Denmark. Minimal inhibitory concentrations (MICs) were determined by the broth microdilution method according to CLSI protocol (CLSI, 2012). Each bacterial strain was diluted to a concentration of 5 × 10^5^ CFU/mL in Luria-Bertani (LB) medium (Statens Serum Institut, Denmark), and was added to a twofold serial dilution of each peptidomimetic in 96-well plates (Nunc Internationals, Rochester, NY, United States). After incubation upon shaking for 18–20 h at 37°C, the MICs were determined as the lowest concentration showing no visible growth. Experiments were performed in triplicates on two different days.

### Time-Kill Kinetics

Planktonic *P. aeruginosa* (0.1 mL with 10^6^ CFU/mL) was added into microtiter wells with LB medium (Hengzhuang et al., 2011). Different concentrations of antimicrobials (in a volume of 0.1 mL) were mixed into different wells, shaken and incubated at 37°C for 0, 1, 2, 4, 8, 12, 24, and 48 h. Samples (0.1 mL) from wells were cultured overnight on LB plates (Staten Serum Institute, Copenhagen, Denmark) and CFU were counted. Experiments were performed in two technical replicates. Killing curves of antimicrobials on planktonic cells were plotted.

### Biofilm Eradication Assay

Biofilm assays were performed by a modified Calgary biofilm device method (Hengzhuang et al., 2011). Isolates were grown overnight in Luria-Bertani (LB) medium. After dilution of this culture to ∼10^7^ CFU/mL, 0.10 mL was transferred to all wells (except for that with the negative control) of a flat-bottom 96-well microtiter plate (catalog no. 269787; Nalgene Nunc International, Rochester, NY, United States). The minimal growth medium (ABTG medium) for biofilm cultivation consisted of 1 mM MgCl_2_, 0.1 mM CaCl_2_, 15.1 mM (NH_4_)_2_SO_4_, 33.7 mM Na_2_HPO_4_, 22 mM KH_2_PO_4_, 51 mM NaCl, 0.01 mM FeCl_3_, 2.5 μg/mL thiamin, and 0.5% glucose. The ABTG medium was refreshed every 24 h in wells for biofilm cultivation. Bacterial biofilms were formed by immersing the pegs of a modified polystyrene microtiter lid (catalog no. 445497; Nunc TSP system) into the biofilm growth plate, followed by incubation at 37°C for 3 days without shaking. The crystal violet staining method was employed to estimate the biofilm formation on the pegs. Peg lids were rinsed three times in sterile water, placed onto flat-bottom microtiter plates containing antimicrobials in twofold dilutions in 0.12 mL of ABTG medium per well (antimicrobial challenge plate), and cultivated for 20 h at 37°C. After antimicrobial incubation, peg lids were again rinsed 3 times in sterile water and placed into antibiotic-free LB in a flat-bottom microtiter plate (biofilm recovery plate) after sonication. To transfer biofilms from pegs to wells, the sonication was performed at 4°C for 2–5 min using Bransonic 220 (80 W, 42 KHz, Branson Co., Shelton, CT, United States). The optical density at 650 nm (OD650) was measured in a microtiter plate reader (Kinetic Microplate Reader, manufactured by Molecular Devices, Novo Biolab, Denmark) before and after incubation at 37°C for 8–12 h. Determination of the minimum biofilm eradication concentration (MBEC) was performed in triplicates on two different days and detected after 24 h recovery, and defined as the concentration of drug that resulted in an increase of OD650 < 0.05.

### *In vitro* Hemolytic Activity

The lysis of human red blood cells (hRBCs) was measured as previously described (Molchanova et al., 2017b). In brief, hRBCs from a freshly drawn type 0+ blood sample were washed with PBS buffer (pH 7.2, 10 mM Tris, 150 mM NaCl), and then centrifuged once at 700 × *g* for 8 min and twice at 1000 × *g* for 8 min each time. Twofold serial dilutions of the peptidomimetics in PBS buffer were added to each well in a round-bottom 96-well plate (Nunc, Thermo Scientific, NY, United States) with a volume of 20 μL in each well. A 1% (v/v) hRBC suspension (80 μL in PBS buffer) was added to reach a total volume of 100 μL in each well. The plate was incubated (37°C) for 1 h, and then the cells were pelleted by centrifugation at 1000 × *g* for 10 min. The supernatant (60 μL) was transferred to a new 96-well plate, and the hemoglobin content was detected by measuring the OD (with a VersaMax Microplate Reader from Molecular Devices, LLC, San Jose, CA, United States) at 414 nm. The OD of samples incubated with melittin (400 μg/mL) defined 100% hemolysis, while the OD of samples incubated with PBS buffer defined 0% hemolysis. Tests were performed twice in triplicates on two different days.

### Cell Culturing

Two different cell lines, HepG2 and NIH 3T3 (both from ATCC, Manassas, VA, United States) were cultured to ∼90% confluence after 21–25 h of growth under standard conditions (5% CO_2_/95% O_2_ at 37°C): Seeding densities for HepG2 and NIH 3T3 were 6.97 × 10^4^ and 6.67 × 10^4^ cells/cm^2^, respectively. HepG2 cells were cultured in Eagle’s Minimal Essential Medium supplemented with 10% (v/v) fetal bovine serum (FBS), 1% (v/v) of non-essential amino acid (NEAA) mixture, and 1 mM sodium pyruvate. NIH 3T3 cells were cultured in Dulbecco’s Modified Eagle’s Medium supplemented with 10% (v/v) newborn calf serum (NCS) (Gibco, Paisley, United Kingdom). All culture media were supplemented with penicillin (100 IU/mL), streptomycin (100 μg/mL), and L-glutamine (2 mM). All cell media and supplements were obtained from Sigma-Aldrich (St. Louis, MO, United States), except serum (Gibco, Paisley, United Kingdom). The 96-well plates were from Corning Costar (Sigma-Aldrich, Brøndby, Denmark).

### Cell Viability Assay

Cell viability assessment was performed on cell monolayers grown to ∼90% confluence in 96-well plates by using the MTS/PMS assay as previously described (Molchanova et al., 2017b). Briefly, the adhered cells were washed with 37°C Hanks’ balanced salt solution (HBSS from Sigma-Aldrich, St. Louis, MO, United States) containing 10 mM HEPES (AppliChem, Darmstadt, Germany), pH 7.4, and exposed for 1 h at 37°C to 100 μL of peptidomimetic dissolved in the medium also used for culturing of each cell line (at concentrations in the range 0–1000 μM). Then the cells were washed twice with 37°C HBSS containing 10 mM HEPES (pH 7.4), and then 100 μL of an MTS/PMS solution, consisting of 240 μg/mL MTS (Promega, Madison, WI, United States) and 2.4 mg/mL PMS (Sigma-Aldrich, Buchs, Switzerland) in HBSS, were added to the cells, which then were incubated for 1 h at 37°C with horizontal shaking (50 rpm) protected from light. A POLARstar OPTIMA plate reader (BMG Labtech, Offenburg, Germany) was used to measure the absorbance at 492 nm. The relative viability was calculated by using 0.2% (w/v) sodium dodecyl sulfate (SDS) as the positive control, while cells exposed to medium without test compound were used as the negative control. Data were obtained in two independent biological replicates performed on separate passages of cells and on separate days with a total number of six replicates.

## Results and Discussion

**FIGURE 1:**
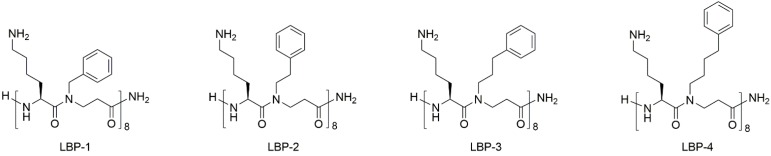
Structures of the peptidomimetics.

Three 16-mer peptidomimetics consisting of alternating cationic lysine and β-peptoid phenylalanine-like hydrophobic residues (Figure 1) were obtained by assembly of dimeric building blocks on a Rink amide resin, followed by cleavage from the linker and final purification by preparative HPLC (Bonke et al., 2008). Within this series (i.e., LBP-2, LBP-3, and LBP-4; Figure 1) the length of the β-peptoid side chains was gradually increased thereby conferring increased hydrophobicity and flexibility. Previously, we have reported a SAR study based on peptidomimetic LBP-1 that showed a general moderate potency against Gram-negative bacteria. Thus, the influence of the length of the cationic side chains was investigated in a series of analogs where ornithine and 2,4-diaminobutyric acid were incorporated instead of lysine (Molchanova et al., 2017b). Although this led to slightly improved activity against Gram-positive bacteria, no improvement in potency was observed toward Gram-negative pathogens including *P. aeruginosa*. As a next step, we introduced an extra carbon into the hydrophobic side chains to yield the 16-mer peptidomimetic LBP-2 (Klodzinska et al., 2018). Interestingly, LBP-2 demonstrated high potency against planktonic *P. aeruginosa*, while the corresponding 12-mer analog displayed more than eightfold lower activity. Therefore, in the present study two novel peptidomimetics LBP-3 and LBP-4 with elongated hydrophobic side chains were designed to further investigate the correlation between hydrophobicity and anti-pseudomonal activity.

Currently, colistin is considered to be the last-resort antibiotic against MDR *P. aeruginosa* infections, however, colistin resistance occurring through a number of various mutations has become critically prevalent (Dosselmann et al., 2017). Therefore, colistin was included as a control when analogs LBP-2, LBP-3, and LBP-4 were tested against a panel of *P. aeruginosa* strains (Table 1), also comprising colistin-resistant clinical isolates and displaying *phoQ* and *pmrB* mutations, obtained from CF patients. PhoPQ and PmrAB systems play important roles in the resistance of *P. aeruginosa* to polymyxin and other cationic antimicrobial peptides (Gutu et al., 2013; Lee et al., 2016). PhoPQ and PmrAB systems regulate the degree of palmitoylation of lipid A in *P. aeruginosa*. In the present study, *P. aeruginosa* strains with *phoQ* and *pmrB* mutations that are involved in resistance to cationic AMPs and colistin were included in the investigation of the killing effect of the series of peptidomimetics.

**Table 1:** Antimicrobial activity of peptidomimetics against *Pseudomonas aeruginosa*, including colistin-resistant clinical isolates from CF patients; and minimal biofilm eradication concentrations (MBECs) against a 3-day-old biofilm.

Compound	MIC (μg/mL)^∗^							MBEC^∗∗^ (μg/mL)
	PAO1	PDO300	PA1016	PA1603	PA77685	PA44638	PA41782	
LBP-2	2	4	4	4	8–16	32	16–32	64
LBP-3	4	4	2	2	8	8–16	16	128
LBP-4	16	16	16	4	16	16	32–64	256
colistin	0.5	0.5	256	>512	32–64	32–64	256	64


*^∗^P. aeruginosa strains: PAO1 – non-mucoid wild type; PDO300 – isogenic mucoid variant of PAO1. Clinical isolates of P. aeruginosa collected from the sputum of CF patients: PA1016 and PA1603 have both phoQ and pmrB mutations; PA77685 and PA44638 have pmrB mutation; PA41782 has phoQ mutation. ^∗∗^MBEC was tested for PAO1 and PDO300 strains for colistin, LBP-2, LBP-3, and LBP-4. The results of MBEC for all tested compounds were similar for both biofilms formed by PAO1 and PDO300 strains. Clinical strains showed poor biofilm formation ability on polystyrene surface system, and therefore these were not used for MBEC testing.*

Analogs LBP-2 and LBP-3 exhibited similar antibacterial activities (Table 1), while LBP-4 displayed a two- to fourfold decreased potency. However, this apparent loss of activity most likely arises from an unfavorable interaction with the medium resulting in partial precipitation due to its high hydrophobicity, which can be correlated to the high percentage of acetonitrile required at peak elution in reversed-phase UHPLC (Table 2; Jahnsen et al., 2015). All analogs displayed similar or slightly lowered activity against colistin-resistant clinical isolates as compared to that seen toward the wild-type strains. Remarkably, the peptidomimetics were more active against colistin-resistant strains PA1016 and PA1603 with both *pmrB* and *phoQ* mutations, while exhibiting four- to eightfold decreased activity against strains possessing either *pmrB* (PA77685, PA44638) or *phoQ* (PA41782) mutation.

**Table 2:** Cell viability (IC_50_), hemolytic activity and hydrophobicity of second-generation peptidomimetics.

Compound	IC_50_ (μg/mL); confidence intervals are stated in brackets				HD_10_ (μM)^∗^	% B^∗∗^
	HepG2		NIH 3T3			
LBP-2	63.1	(59.7–67.0)	181.0	(153.0–217.0)	>300	37.8
LBP-3	24.3	(20.8– 8.3)	65.3	(60.4–70.6)	<4.7 (81%)	43.8
LBP-4	33.7	(31.2–37.9)	104.0	(95.4–113.0)	<4.7 (100%)	49.3


*^∗^HD_10_ designates the concentration of peptidomimetic that results in hemolysis of 10% of human red blood cells; values are also stated in brackets as percent of hemolysis at 4.7 μM. ^∗∗^Hydrophobicity is indicated by the percentage of acetonitrile (%B) at the peak elution time on analytical reversed-phase HPLC.*

Anti-biofilm potency was detected against a mature biofilm (3-days-old) grown on peg lids immersed into media in a microtiter plate. Though none of the analogs exhibited improved anti-biofilm activity as compared to that of colistin, it is noticeable that the least hydrophobic analog was the only peptidomimetic that displayed potency similar to that of colistin with respect to biofilm eradication (Table 1).

In order to assess the potential therapeutic utility of the peptidomimetics (e.g., in animal studies), their hemolytic properties as well as effects on cell viability of HepG2 and NIH 3T3 cell lines were determined (Table 2). Expectedly, hydrophobicity was found to be closely correlated to the hemolytic activity of the compounds. Nevertheless, it was somewhat surprising that incorporation of one additional carbon in the hydrophobic side chains resulted in such a dramatic increase in hemolytic properties (i.e., ∼100-fold lower HD_10_ value). The overall cytotoxicity profiles also reflected this, but to a lower degree, since the IC_50_ values for analog LBP-3 were less than threefold lower than those of LBP-2, whereas the relatively higher values for LBP-4 can be ascribed to limited solubility in the medium (seen as a turbidity).

**FIGURE 2:**
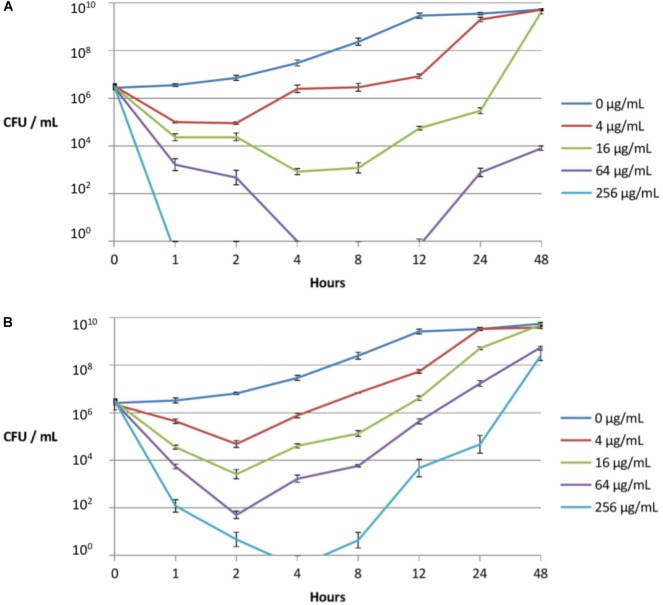
Time-kill kinetics of peptidomimetic LBP-2 **(A)** and colistin **(B)** against a clinical resistant strain (PA1603; with phoQ and pmrAB mutations) isolated from a CF lung. Error bars indicate ± SD.

Since compound LBP-2 displayed high potency and the most favorable cell selectivity it was selected for further investigation. As MIC values mainly assess antibiotic efficacy, but do not provide details on the dynamics of the drug-bacteria interaction, time-kill kinetics of LBP-2 and colistin were determined (during 48 h) against the colistin-resistant strain PA1603, isolated from a CF patient (Figure 2).

PhoPQ and PmrAB system loss-of-function mutations can contribute to high-level colistin resistance in clinical strains of *P. aeruginosa* (Miller et al., 2011; Moskowitz et al., 2012). The disruption of chromosomal *phoQ* in the presence of an intact *phoP* allele stimulates 4-amino-L-arabinose addition to lipid A and induces transcription from the promoter of the *pmrH* (*arnB)* operon. The expression of mutant *pmrB* alleles induces transcription from the promoter of the *arnB* operon and further stimulates addition of 4-amino-L-arabinose to lipid A. Such lipid A modifications are known to play a key role in colistin resistance (Miller et al., 2011; Gutu et al., 2013).

As seen from the time-kill curves (Figure 2), peptidomimetic LBP-2 at the highest concentration (256 μg/mL) gave rise to complete killing within 1 h with no regrowth, whereas colistin failed to exhibit complete killing at the same concentration with ensuing regrowth after 4 h. In addition, regrowth occurs only after a lag period of 4–8 h when treating with 16 or 64 μg/mL of peptidomimetic, whereas regrowth upon treatment with colistin at these concentrations appears to occur immediately after reaching the maximal initial killing. This indicates that adaption to LBP-2 might involve a different resistance mechanism as compared to that for colistin, which in turn infers that LBP-2 and colistin act via different killing mechanisms. Previous studies on a related subclass of α-peptide/β-peptoid hybrids indicated a mode of action involving a general membrane-disruptive process (Hein-Kristensen et al., 2011).

In order to investigate the stability of peptidomimetic LBP-2 toward proteolytic degradation, it was exposed to the versatile degradation enzyme pronase (a commercial protease mixture isolated from *Streptomyces griseus*) for 48 h. As seen from Supplementary Figure S1 compound LBP-2 was not degraded by pronase, and therefore it is considered resistant to proteolysis over a period of 24 h. This is in accordance with a previous study where all-L peptides were found to be prone to enzymatic degradation, while the peptoid analogs were essentially unaffected (Miller et al., 1994). Other peptidomimetics such as α/β-peptides, α-peptoids, β-peptoids and peptide-peptoid hybrids have likewise been demonstrated to possess similar stability toward proteolysis (Schmitt et al., 2007; Jahnsen et al., 2012).

Several studies have shown that cationic peptides and peptidomimetics display high bactericidal activity against planktonic *P. aeruginosa* cells, as well as an ability to prevent biofilm formation. To date, LL-37 is the most studied peptide capable of inhibiting *P. aeruginosa* biofilm formation at a concentration of 0.5 μg/mL, which is 16-fold below its MIC value against planktonic *P. aeruginosa* (Overhage et al., 2008), but also the peptides IDR-1018 (at 0.8 μg/mL), DJK-5 and DJK-6 (at 10 μg/mL) exhibit activity against *P. aeruginosa* biofilms (De Brucker et al., 2014; de la Fuente-Nunez et al., 2015; Mansour et al., 2015). Other examples of anti-biofilm compounds comprise three α-helical peptides that were able to reduce biofilm formation at sub-inhibitory concentrations, while two of them showed biofilm-reducing activity equal to that of tobramycin (Pompilio et al., 2012). In addition, several peptidomimetics have also been found to have similar anti-biofilm properties, e.g., the group of Barron showed that a peptoid and its lipidated shorter analog were less effective in eradicating established biofilms than in preventing their formation. Thus, these compounds were capable of killing planktonic cells that could otherwise contribute to establishment of biofilm (Kapoor et al., 2011). Another study on lipopeptides demonstrated the same trend, however, only the least hydrophobic cationic lipopeptides were able to penetrate deep into the innermost layers of the biofilm matrix, hence exhibiting high killing efficacy (Sanchez-Gomez et al., 2015). In another study, the peptide-peptoid hybrid HDM-4 exhibited strong anti-biofilm activity at sub-MIC level (Jahnsen et al., 2013). Noticeably, most of the mentioned peptides and peptidomimetics display biofilm-inhibitory activity rather than an ability to eradicate established biofilms, which is the case only for a few compounds.

## Conclusion

Our data for the LBP series support the trend that, albeit the peptidomimetics display high activity against planktonic cells, they lack efficacy toward established biofilms. Additionally, in accordance with previous observations, a correlation between relatively low hydrophobicity and improved anti-biofilm activity was demonstrated. Compound LBP-2 possessed the most promising cytotoxicity profile and exhibited rapid bactericidal effect against a colistin-resistant *P. aeruginosa* strain. Moreover, peptidomimetic LBP-2 exhibited potent activity against a colistin-resistant *P. aeruginosa* strain with *phoQ* and *pmrB* mutations as compared to that of colistin (i.e., 4 vs. >512 μg/mL). Therefore, the mechanisms of action of compound LBP-2 and colistin appear to be different, inferring that peptidomimetic LBP-2 may not bind or binds in a different way to LPS in the outer membrane of *P. aeruginosa*. In addition, colistin and LBP-2 exerted similar potency in anti-biofilm inhibition (64 μg/mL). Compound LBP-2 displayed a faster and more efficient killing kinetics after just 1 h at 256 μg/mL, while killing was slower and regrowth was observed already after 4 h for bacteria exposed to colistin at the same concentration. Peptidomimetic LBP-2 proved to be stable toward enzymatic degradation, but exhibited some cytotoxicity.

## Ethics Statement

The clinical isolates were obtained from Department of Clinical Microbiology, University Hospital of Copenhagen, Denmark. All bacterial strains were revived from freeze storage, while blood was obtained from a donor who was fully informed about the study and purpose and a written consent has been received. According to the Danish Science Ethics Committee, the study did not need approval from the Committee (Protocol nr H-2-2013-FSP45).

## Author Contributions

NM synthesized and tested the compounds, as well as drafted the manuscript. HW tested the compounds. All authors contributed to the design of compounds and experiments as well as manuscript revision, reading and approval of the submitted version.

## Conflict of Interest Statement

The authors declare that the research was conducted in the absence of any commercial or financial relationships that could be construed as a potential conflict of interest.
