# Supplementary material for: Antimicrobial Activity of α-Peptide/β-Peptoid Lysine-Based Peptidomimetics Against Colistin-Resistant Pseudomonas aeruginosa Isolated From Cystic Fibrosis Patients
Source: Front Microbiol. 2019 Feb 20;10:275. doi: 10.3389/fmicb.2019.00275 (PMC6391360; doi:10.3389/fmicb.2019.00275)
Supplement: Supplementary file 1 [file Data_Sheet_1.PDF]

Supplementary Material

Antimicrobial activity of α-peptide/β-peptoid lysine-based peptidomimetics against colistin-resistant *Pseudomonas aeruginosa* isolated from cystic fibrosis patients

Natalia Molchanova, HengZhuang Wang, Paul R Hansen, Niels Høiby, Hanne M Nielsen, Henrik Franzyk*

*** Correspondence:**
Corresponding author
henrik.franzyk@sund.ku.dk

1 NMR of building blocks:

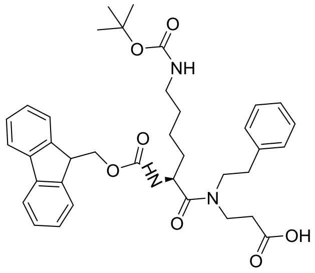

^{1}H NMR (600 MHz, MeOD) δ 7.77 (d, J = 7.5 Hz, 2H), 7.69 – 7.61 (m, 2H), 7.36 (t, J = 7.5 Hz, 2H), 7.32 – 7.07 (m, 9H), 4.59 – 4.31 (m, 3H), 4.28 – 4.08 (m, 1H), 3.77 – 3.49 (m, 3H), 3.49 – 3.34 (m, 1H), 3.11 – 2.77 (m, 4H), 2.75 – 2.47 (m, 2H), 1.40 (s, 9H), 1.67 – 1.16 (m, 4H).

^{13}C NMR (150 MHz, MeOD) δ 175.3, 174.6, 174.5, 174.5, 158.5, 158.5, 158.4, 158.3, 145.3, 145.2, 145.2, 142.6, 140.2, 139.6, 138.9, 130.1, 130.0, 129.9, 129.7, 129.5, 129.2, 128.8, 128.2, 128.1, 127.7, 127.4, 126.3, 126.2, 126.2, 120.9, 79.8, 67.9, 67.8, 52.4, 52.3, 51.4, 45.0, 44.2, 41.0, 36.3, 34.6, 34.5, 33.1, 33.0, 32.9, 30.6, 28.8, 24.0, 21.5.

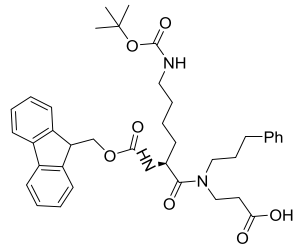

^{1}H NMR (600 MHz, MeOD) δ 7.78 (dd, J = 11.1, 7.6 Hz, 2H), 7.71 – 7.59 (m, 2H), 7.41 – 7.33 (m, 2H), 7.33 – 7.08 (m, 9H), 4.59 – 4.26 (m, 3H), 4.20 (t, J = 6.9 Hz, 1H), 3.80 – 3.59 (m, 1H), 3.55 – 3.33 (m, 2H), 3.06 – 2.93 (m, 2H), 2.77 – 2.40 (m, 4H), 2.11 – 1.91 (m, 1H), 1.88 – 1.07 (m, 7H), 1.42 (s, 9H).

^{13}C NMR (150 MHz, MeOD) δ 175.4, 174.7, 174.6, 174.5, 158.5, 158.4, 145.3, 145.2, 143.0, 142.6, 142.3, 138.9, 129.9, 129.6, 129.5, 129.4, 129.3, 129.2, 128.8, 128.2, 127.1, 126.9, 126.3, 126.3, 126.2, 126.2, 120.9, 120.9, 79.9, 67.9, 65.0, 52.5, 52.5, 49.2, 48.5, 47.3, 44.9, 44.7, 44.2, 41.0, 34.7, 34.2, 33.8, 33.4, 33.0, 32.9, 31.6, 30.6, 30.5, 30.4, 28.8, 28.7, 24.1, 21.5.

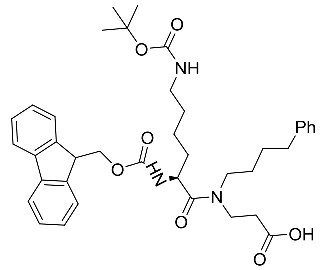

^{1}H NMR (600 MHz, MeOD) δ 7.79 (dd, J = 7.6, 3.9 Hz, 2H), 7.70 – 7.61 (m, 2H), 7.38 (td, J = 7.4, 2.8 Hz, 2H), 7.36 – 7.27 (m, 2H), 7.20 (q, J = 7.1 Hz, 2H), 7.15 (t, J = 7.6 Hz, 2H), 7.13 – 7.08 (m, 1H), 4.59 – 4.42 (m, 1H), 4.43 – 4.27 (m, 2H), 4.23 – 4.11 (m, 1H), 3.77 – 3.55 (m, 1H), 3.55 – 3.35 (m, 2H), 3.10 – 2.98 (m, 2H), 2.79 – 2.43 (m, 4H), 1.77 – 1.21 (m, 10H), 1.41 (s, 9H).

^{13}C NMR (150 MHz, MeOD) δ 175.3, 174.6, 174.6, 174.3, 158.5, 158.4, 158.3, 145.3, 145.3, 145.2, 145.2, 143.5, 143.2, 142.6, 129.9, 129.4, 129.4, 129.4, 129.3, 129.2, 128.8, 128.2, 126.8, 126.7, 126.3, 126.3, 126.2, 120.9, 120.9, 79.8, 67.9, 52.5, 52.3, 46.9, 44.5, 44.1, 41.0, 36.5, 36.4, 36.3, 34.6, 33.2, 33.0, 30.6, 29.8, 29.7, 29.6, 28.8, 28.7, 28.0, 24.1, 24.0.

2 Description of peptidomimetics:

Peptidomimetic LBP-2. Charge: +9. t_R = 5.95 min, purity 97.85% Gradient: 10-60% B over 10 min. B = 95% MeCN + 0.1% TFA. HRMS: calcd for $[M+4H]^{4+}$ 611.6538, found 611.6532; ΔM = 1 ppm.

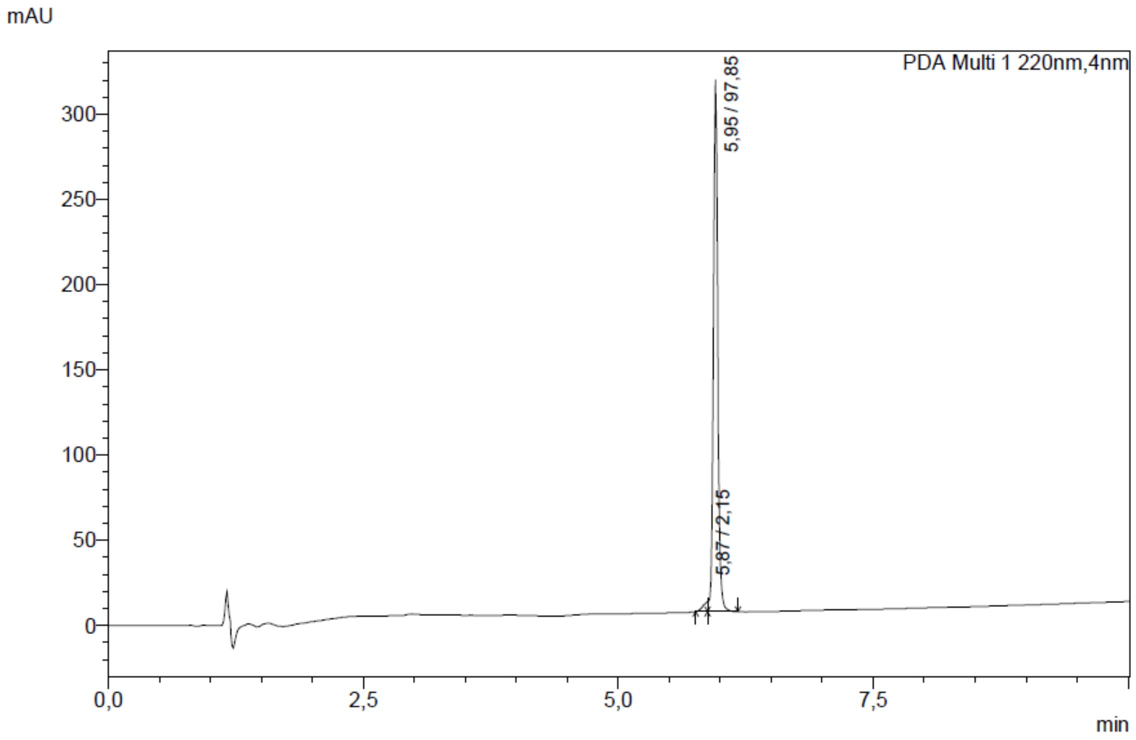

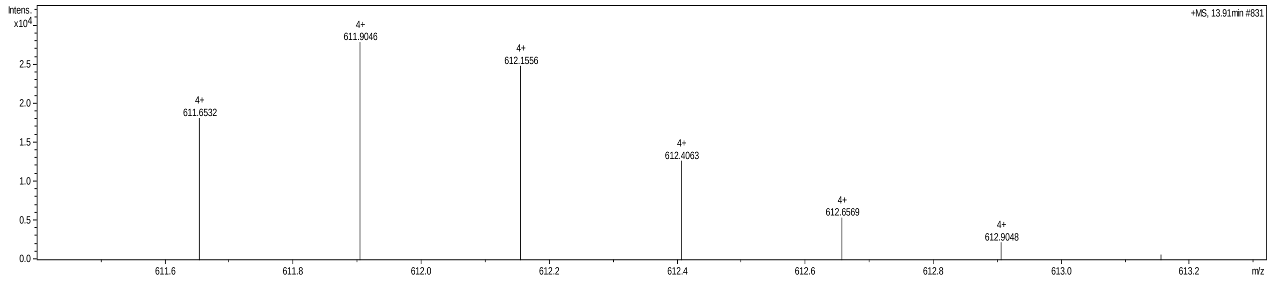

Peptidomimetic LBP-3. Charge: +9. t_R = 8.30 min, purity 99.68% Gradient: 10-60% B over 10 min. B = 95% MeCN + 0.1% TFA. HRMS: calcd for $[M+6H]^{6+}$ 426.7927, found 426.7939; ΔM= 2.8 ppm.

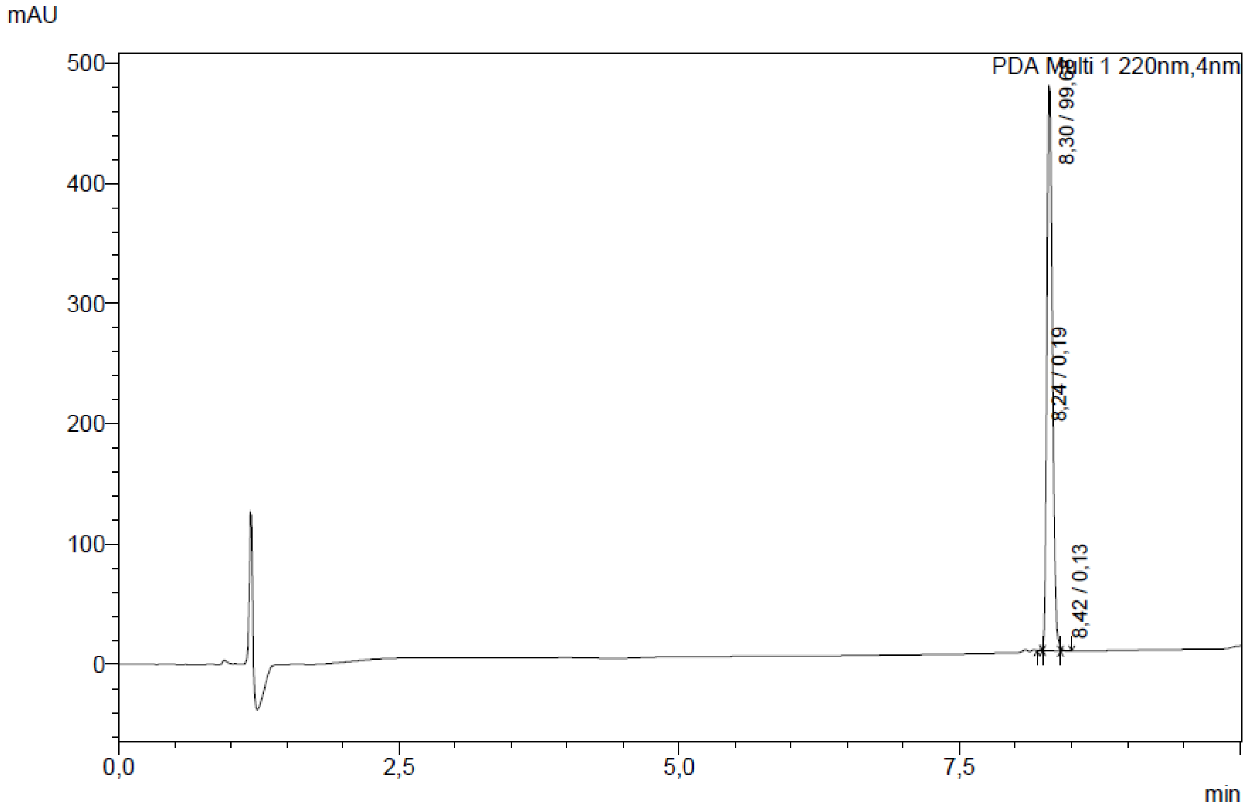

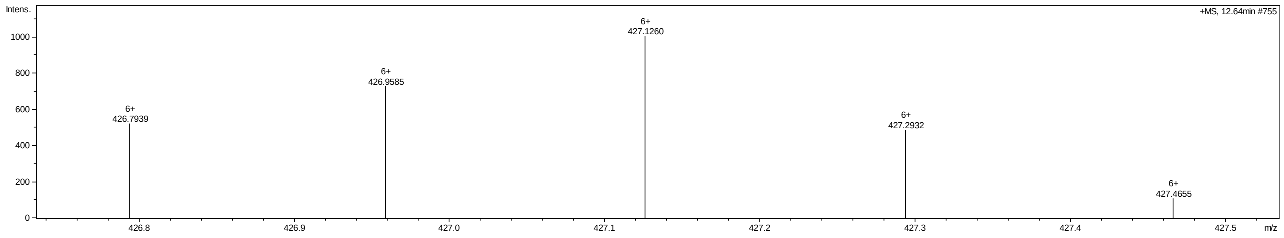

Peptidomimetic LBP-4. Charge: +9. t_R = 8.27 min, purity 98.10% Gradient: 10-60% B over 10 min. B = 95% MeCN + 0.1% TFA. HRMS: calcd for $[M+6H]^{6+}$ 445.4802, found 445.4790; ΔM= 2.7 ppm.

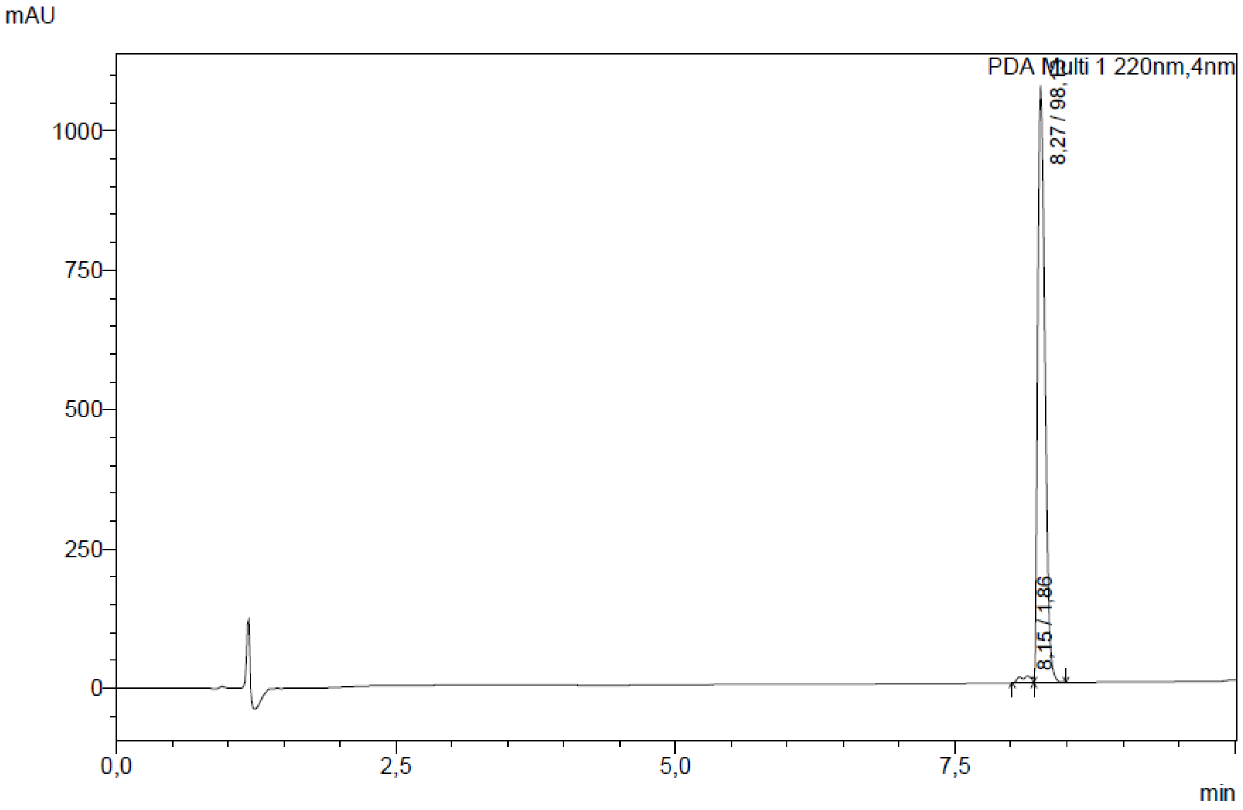

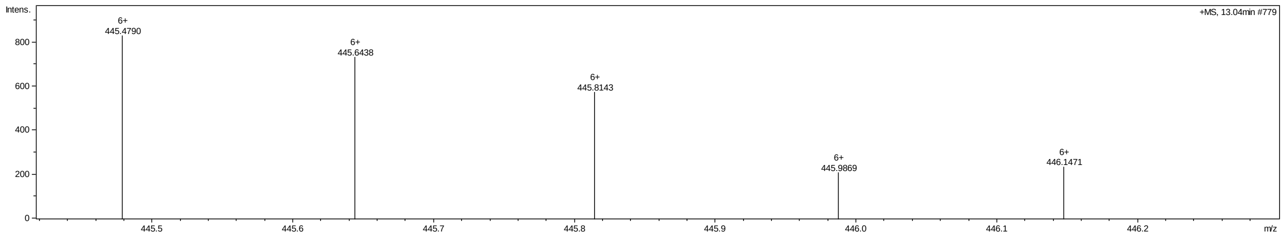

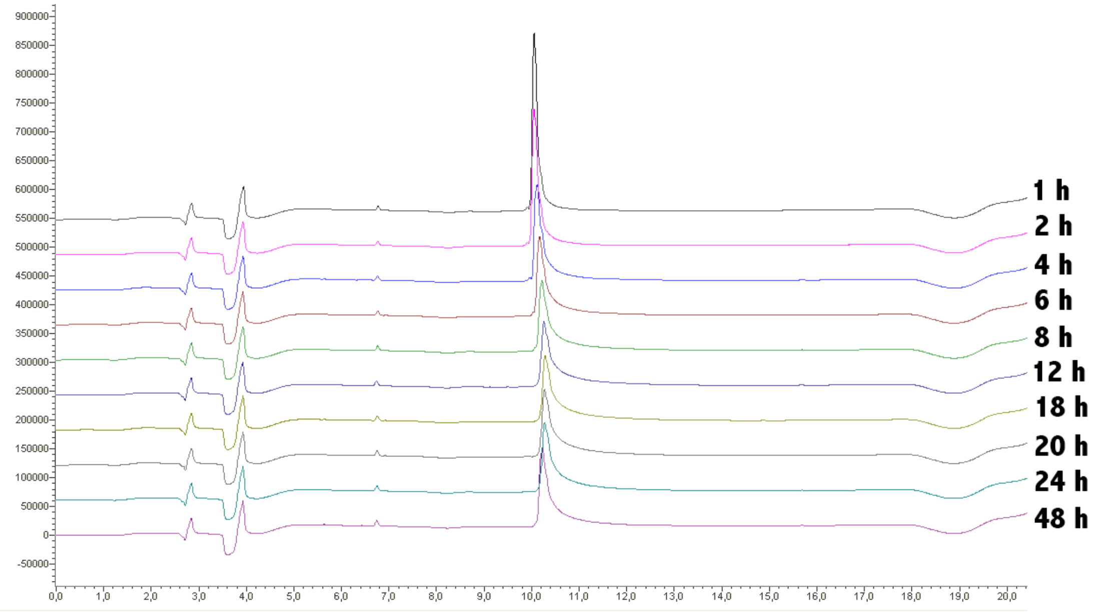

Figure S1. Stability testing of LBP-2 in presence of pronase.
